# The Futile Cycling of Hexose Phosphates Could Account for the Fact That Hexokinase Exerts a High Control on Glucose Phosphorylation but Not on Glycolytic Rate in Transgenic Potato (*Solanum tuberosum*) Roots

**DOI:** 10.1371/journal.pone.0053898

**Published:** 2013-01-28

**Authors:** Éric Claeyssen, Sonia Dorion, Audrey Clendenning, Jiang Zhou He, Owen Wally, Jingkui Chen, Evgenia L. Auslender, Marie-Claude Moisan, Mario Jolicoeur, Jean Rivoal

**Affiliations:** 1 Institut de Recherche en Biologie Végétale, Université de Montréal, Montréal, Québec, Canada; 2 Department of Chemical Engineering, École Polytechnique de Montréal, Montréal, Québec, Canada; Friedrich-Alexander-University Erlangen-Nurenberg, Germany

## Abstract

The metabolism of potato (*Solanum tuberosum*) roots constitutively over- and underexpressing hexokinase (HK, EC 2.7.1.1) was examined. An 11-fold variation in HK activity resulted in altered root growth, with antisense roots growing better than sense roots. Quantification of sugars, organic acids and amino acids in transgenic roots demonstrated that the manipulation of HK activity had very little effect on the intracellular pools of these metabolites. However, adenylate and free Pi levels were negatively affected by an increase in HK activity. The flux control coefficient of HK over the phosphorylation of glucose was measured for the first time in plants. Its value varied with HK level. It reached 1.71 at or below normal HK activity value and was much lower (0.32) at very high HK levels. Measurements of glycolytic flux and O_2_ uptake rates demonstrated that the differences in glucose phosphorylation did not affect significantly glycolytic and respiratory metabolism. We hypothesized that these results could be explained by the existence of a futile cycle between the pools of hexose-Ps and carbohydrates. This view is supported by several lines of evidence. Firstly, activities of enzymes capable of catalyzing these reactions were detected in roots, including a hexose-P phosphatase. Secondly, metabolic tracer experiments using ^14^C-glucose as precursor showed the formation of ^14^C-fructose and ^14^C-sucrose. We conclude that futile cycling of hexose-P could be partially responsible for the differences in energetic status in roots with high and low HK activity and possibly cause the observed alterations in growth in transgenic roots. The involvement of HK and futile cycles in the control of glucose-6P metabolism is discussed.

## Introduction

Glycolysis is central to cell metabolism as it oxidizes hexoses to generate ATP and produces building blocks for various biosynthetic pathways [1]. Hexokinase (HK, EC 2.7.1.1) is a glycolytic enzyme that catalyzes the irreversible ATP-dependent phosphorylation of hexoses such as glucose (Glc) and fructose (Fru). Its specificity for several hexose substrates [2]–[4] makes this enzyme a gateway to carbohydrate metabolism, and a potentially major controlling step of the glycolytic pathway [2], [5], [6]. It has been unambiguously demonstrated that HK also functions as a sugar sensor in Eukaryotes [7], [8] and that the function of HK in plant sugar sensing and signaling is distinct and independent from its catalytic activity [9].

HK has been identified as an important site of glycolytic flux control in several eukaryotic organisms. Thus, the flux control coefficient (FCC) of HK over glycolysis exceeds 0.7 in mammalian erythrocytes, liver, heart, pancreas, insulinoma and muscle cells [10]–[15]. In yeast (*Saccharomyces cerevisiae*), mutant studies have implicated HK in the control of glycolytic flux via its inhibition by trehalose-6-phosphate (T6P) [16], [17]. It has been proposed that yeast glycolysis operates according to an autocatalytic principle in which ATP is consumed to drive Glc catabolism before being replenished by subsequent metabolism. HK inhibition by T6P would restrict Glc flux into glycolysis, thereby preventing a stall during an abrupt increase in Glc supply [17]. Accordingly, there is evidence to suggest that the relatively low (0.2 to 0.5) FCC of HK over yeast glycolysis may be due to its potent inhibition by T6P [18], [19]. With respect to plants, the characterization of several purified HKs has led to the conclusion that they are not regulated by T6P [3], [20]. The measurement of the FCC of HK over Glc metabolism still awaits measurement and the question of whether and how HK may control glycolytic flux is still unanswered [2]. Nonetheless, indirect evidence suggests an impact of HK on C metabolism. For example, increasing HK activity levels in tomato (*Solanum lycopersicum*) plants resulted in growth inhibition, leaf senescence, perturbed carbon metabolism, reduced photosynthesis and stomatal conductance [21]–[23]. Increases in HK catalytic activity have also been linked to the perturbation of energy metabolism in tomato fruit [22]. In connexion to this, it is interesting to note that HK can possibly also take part in futile cycles occurring between sucrose (Suc) and hexoses and between hexose and hexose-P pools [24], [25]. Altogether, the Suc and Glc/Glc-P substrate futile cycles may consume 40–80% of the ATP generated in maize (*Zea mays*) root tips [25]–[27]. Interestingly, it has been shown that plant glycolytic flux is controlled, at least in part, by ATP demand [28], [29]. Suc and Glc/Glc-P cycles, therefore, have been proposed to raise glycolytic flux through increased energy demand [25], [28]. These findings suggest new possibilities for plant HK to contribute to glycolytic flux control via Suc and Glc/Glc-P cycling and modulation of the cell’s energy status [2].

Plant roots depend on the supply of C from other plant parts for their central metabolism. In that view, we aimed at clarifying the relevance of HK to primary metabolism and to the control of glycolytic flux in this heterotrophic organ. Potato (*Solanum tuberosum*) roots were altered in their HK activity by transformation with an HK cDNA in sense or antisense orientation. We show that altering root HK activity levels impacts on growth rate and that HK has a high FCC over Glc phosphorylation but does not control glycolytic flux or respiration rate. We also found evidence for a root glucose-6P phosphatase (G6Pase) activity that could be involved in cycling glucose-6P (G6P) back to the hexose pool. Labeling studies conducted with [U-^14^C]Glc also support the existence of cycling between Glc and Suc. Based on these data, we propose that the tight control of G6P metabolism in roots has major implications in the regulation of glycolysis, respiration and growth.

## Materials and Methods

### Biological Materials and Chemicals

Potato plants (*Solanum tuberosum*, cv. Russet Burbank) used for transformation were grown in growth chambers at 23°C with 12 h light period until the age of 4 to 6 weeks. *Agrobacterium rhizogenes* wild-type strain A4 [30] was maintained as described [31]. All buffers, chemicals, reagents and commercial enzymes were of analytical grade and purchased from Sigma Chemical Co. (St-Louis, MO), Invitrogen (Burlington, ON, Canada) or Fisher Scientific (Nepean, ON, Canada), unless otherwise mentioned. PD10 columns were from G.E. Healthcare (Baie d’Urfé, QC, Canada). Fractogel EMD DEAE-650 (S) was from VWR (Mississauga, ON, Canada). Dowex AG 50W-X8 (H^+^) and Dowex AG 1-X8 (formate) resins were from Bio-Rad Laboratories Inc. (Mississauga, ON, Canada**)**. Restriction enzymes were from MBI-Fermentas and Invitrogen Canada Inc. (Burlington, ON, Canada). Radiolabeled tracers 2-deoxy[U-^14^C]Glc ([U-^14^C]DOG), 2-deoxy[1-^3^H]Glc ([1-^3^H]DOG), [U-^14^C]Glc and [U-^14^C]Suc were purchased from Moravek Biochemicals Inc. (Brea, CA, USA).

### Construction of Plasmids and Transformation

One sense and three antisense constructs were prepared using *ScHK2* which shares over 97% homology with potato StHK2 [20] and was therefore expected to be used successfully in an antisense strategy. Sense and antisense *ScHK2* constructs were inserted into the binary vector pGA643 under the control of the cauliflower mosaic virus 35S promoter. The sense construct was obtained by digesting the *ScHK2* cDNA from pBK-CMV [20] with *Cla*I and *Hinc*II. The resulting 1850 bp fragment was cloned into *Cla*I:*Hpa*I-digested pGA643 plasmid. Digestion of the *ScHK2* cDNA with *Xba*I and *Eco*RV, and with *Xba*I and *Hinc*II, generated antisense constructs 1 (606 bp) and 3 (1829 bp), respectively. Each construct was cloned into *Xba*I:*Hpa*I-digested pGA643 plasmid. For preparation of antisense construct 2, pGA643 was digested with *Hpa*I and dephosphorylated with 150 U of bacterial alkaline phosphatase (Invitrogen) for 1 h at 65°C. The *ScHK2* cDNA was then digested with *Eco*RV and *Hinc*II into a 1212 bp fragment that was cloned into the prepared pGA643 vector. Presence and correct orientation of the inserts were confirmed by restriction digests and sequencing. Root clones transformed with an empty vector were named Ctrl, whereas sense (S) and antisense (AS) clones were transformed with engineered plasmids. The first digit in AS clones identified the constructs described above. The last two digits identify individual clones. Freshly cut potato stems were transformed as described by [32] for tomato petioles, except that MS medium [33] was supplemented here with 3% (w/v) Suc. Roots were subcultured every 3 weeks on MS medium containing 0.4% (w/v) Phytagel.

### Growth Measurement

Single root tips, 0.7 cm in length, were laid in the center of Petri plates containing 15 mL of solid MS medium, and left to grow in the dark. After 14 d, the root systems were scanned and analyzed with the WinRhizo software (Régent Instruments Inc., Québec, QC, Canada) for measurement of total root length, tip number and mean diameter.

### HK Extraction from Potato Roots and Activity Assay

Root clones were subcultured in 250 mL Erlenmeyer flasks containing 25 mL of liquid MS medium and agitated continuously at 145 rpm on a gyratory shaker, at 23°C. Roots were harvested, frozen with liquid N_2_ and stored at −80°C until used. All subsequent steps were carried out at 4°C. Roots were ground with a mortar and pestle using a 2∶1 (mL extraction buffer g^−1^ FW) ratio in a buffer containing 100 mM Tris-HCl, pH 8.0, 10 mM KCl, 10 mM MgCl_2_, 5 mM DTT, 1 mM EDTA, 1 mM EGTA, 1 mM ε-CA, 1 mM benzamidine, 5% (w/v) insoluble PVPP, 0.1% (v/v) Triton X-100, 10% (v/v) glycerol and 1 mM PMSF. Homogenates were centrifuged for 15 min at 12,000×*g*. The resulting supernatant was desalted on PD10 columns pre-equilibrated with desalting buffer (20 mM Tris-HCl, pH 8.2, 0.5 mM MgCl_2_, 1 mM DTT and 10% (v/v) glycerol). HK activity assays were conducted on desalted extracts as in [20]. Enzymes specific activities are expressed in U mg^−1^ protein (1 U of activity corresponds to the appearance of reaction product at the rate of 1 µmol min^−1^ at 30°C). Protein concentration was determined according to [34], using BSA as standard and the Bio-Rad protein assay reagent (Bio-Rad, Mississauga, ON, Canada).

### Extraction and Assays of Other Primary Metabolism Enzymes

All steps were carried out at 4°C. Roots were ground with a mortar and pestle using a 2∶1 (mL extraction buffer g^−1^ FW) ratio in a buffer containing 30 mM Hepes-KOH, pH 7.5, 100 mM KCl, 5 mM MgCl_2_, 5 mM DTT, 1 mM EDTA, 1 mM EGTA, 5 mM ε-CA, 1 mM benzamidine, 1 mg L^−1^ leupeptin, 5% (w/v) insoluble PVPP, 0.1% (v/v) Triton X-100, 4% (w/v) polyethylene glycol 8000, 20% (v/v) glycerol and 2 mM PMSF. Homogenates were centrifuged for 15 min at 12,000×*g*. Clear supernatants were desalted on PD10 columns pre-equilibrated with desalting buffer (30 mM Hepes-KOH, pH 7.5, 100 mM KCl, 5 mM MgCl_2_, 1 mM DTT, 1 mM EDTA, 1 mM EGTA, 4% (w/v) polyethylene glycol and 20% (v/v) glycerol). The pellet was washed four times and incubated overnight with 20 mM MES-KOH, pH 6 containing 1 M NaCl to release invertase (INV, EC 3.2.1.26) activity bound to the cell wall. Except when otherwise indicated, enzymes were measured using coupled assays based on production or consumption of NADH at 340 nm, at 30°C. Phospho*enol*pyruvate carboxylase (PEPC, EC 4.1.1.31) was assayed as in [35]. Assays of pyruvate kinase (PK, EC 2.7.1.40) and phospho*enol*pyruvate phosphatase (PEPase, EC 3.1.3.60) were modified from [36] and from [37]. Assays of phosphoglucomutase (PGM, EC 5.4.2.2), sucrose synthase (SuSy, EC 2.4.1.13), ADPGlc pyrophosphorylase (AGPase, EC 2.7.7.27) and UDPGlc pyrophosphorylase (UGPase, EC 2.7.7.9) were adapted from [38]. PhosphoGlc isomerase (PGI, EC 5.3.1.9) assay was modified from [39]. Aldolase (ALD, EC 4.1.2.13) was assayed as in [40]. Assays for ATP-dependent phosphofructokinase (PFK, EC 2.7.1.11), fructose-1,6-bisphosphatase (FBPase, EC 3.1.3.11) and pyrophosphate-fructose-6-phosphate 1-phosphotransferase (PFP, EC 2.7.1.90) were modified from [41]. Assay for triosephosphate isomerase (TPI, EC 5.3.1.1) was from [42]. Assay of NAD(P)-dependent glyceraldehyde-3-phosphate dehydrogenase (GAPDH, EC 1.2.1.12 and EC 1.2.1.13) was modified from [43]. INV actvity was assayed in desalted extracts at pH 5 (Acid INV), at pH 7 (Neutral INV) and at pH 5 after desorption from the cell wall fraction (Cell wall INV) by an enzymatic assay of hexoses released from hydrolysis of Suc [29]. Assays were initiated by addition of enzyme preparation and corrected for background activity by omitting substrate from the reaction mixture. Hexose-P phosphatase activity was assayed using G6P as substrate (G6Pase, EC 3.1.3.9) in a coupled assay with purine nucleoside phosphorylase (EC 2.4.2.1) by following at 360 nm the phosphorylation of methylthioguanosine using Pi released from G6P [44]. Under these conditions, the optimum pH of G6Pase was 7.5. The assay buffer contained 50 mM Tris-Cl, pH 7.5, 1 mM MgCl_2_, 0.1 mM NaN_3_ and 200 µM methylthioguanosine. The amount of Pi released in the assay was established by comparison with a Pi standard curve done under the same conditions. For all enzymes studied, reaction rates were linear with time and proportional to the amount of enzyme added to the assay. The stoichiometry of the reactions was taken in account in activity calculations.

### SDS/PAGE, Immunoblot Analysis of HK

SDS/PAGE analysis was performed on 12% (w/v) acrylamide gels according to [45]. Immunodetection analysis was performed with affinity-purified rabbit immune serum (1/15 dilution) raised against a truncated version of ScHK2 protein [20]. Immunoblots incubated with the pre-immune serum gave negative results (data not shown).

### Analytical Anion-exchange Chromatography of HK Isoforms

Roots grown on solid MS medium were extracted and clarified as described above for HK activity assays except that extraction buffer contained 50 mM Tris-HCl, pH 8.0, and 5 mM MgCl_2_. Clarified extracts (2 mg protein) were then desalted and loaded onto a column (1×8 cm) of Fractogel EMD DEAE-650 (S) as described in [20]. One mL fractions were collected and assayed. Typically, HK activity was resolved as two peaks eluting between 125 and 200 mM KCl. An additional FK activity peak eluted between 225 and 300 mM KCl. Representative activity profiles are shown.

### O_2_ Uptake

O_2_ uptake rates were measured as described previously [46] using a Clark type O_2_ electrode from Qubit Systems (Kingston, ON, Canada) controlled by the Logger Pro 3.2 software. Root samples (approximately 100 mg FW) were inserted into the chamber which was thermostated at 25°C and contained 4 mL MS medium. O_2_ uptake rates were recorded for 5 min.

### Metabolite Measurements

Roots were flash frozen with liquid nitrogen, stored at −80°C and processed as described below, according to the type of analysis. Data were corrected for recovery. For the spectrophotometric determination of hexose-P, roots were processed and analyzed as described by [47]. Starch was hydrolyzed and assayed enzymatically as Glc [48]. Adenylates were determined by luminescence [49] using a Molecular Devices Spectramax L microplate luminometer. For Pi determination, roots were extracted in 1% (v/v) glacial acetic acid using a ratio of 20 mL/g FW. After clarification of the extract (15 min centrifugation at 16,000 *g*), the supernatant was used for Pi quantification using the procedure of [50]. For HPLC analysis, a precisely weighted amount of approximately 0.5 g FW frozen material was extracted at 75°C in 10 mL of 80% (v/v) ethanol. Extracts were resolubilized in 3 mL H_2_O and fractionated by ion exchange on a column of Dowex AG 50W-X8 (H^+^) and a column of Dowex AG 1-X8 (formate) arranged in tandem [51]. Sugars, organic acids and amino acids eluted in neutral, anionic and cationic fractions, respectively. An aliquot of each fraction was evaporated to dryness and subsequently analyzed by HPLC as previously described [46].

### *In vivo* NMR Spectroscopy

Root samples (approximately 0.5 g) were placed in a modified screw cap 10 mm NMR tube. Roots were perfused by oxygenated MS medium (10% (v/v) [52]. D_2_O was used to lock NMR signal) free of Pi and paramagnetic ions at a flow rate of 1.7 ml/min. A microcapillary tube (ID = 0.199 mm) containing 2M methylphosphonic acid was inserted into inlet tube and used for chemical shift (30.60 ppm down shift from 85% orthophosphonic acid) and concentration reference. ^31^P NMR spectroscopy was performed on a Varian Unity Inova 400 MHz NMR spectrometer with 10 mm Broadband probe (Varian inc. USA). NMR data were recorded after 1 h of perfusion. A ^31^P spectrum was acquired at 161.839 MHz at 298.15 K with a delay time of 0.5 s and 45° pulse. The acquisition time was set to 0.69 s and spectral width was 20 KHz. The spectra were acquired with 3008 scans corresponding to a total acquisition time of 1 h. The relative quantification of vacuolar Pi and cytoplasmic Pi was achieved by comparing the peak areas of vacuolar Pi and cytoplasmic Pi. Vacuolar and cytoplasmic pHs were determined using a standard curve of the ^31^P NMR chemical shifts for Pi recorded under different pH conditions [52].

### Synthesis of Radiolabeled Standard 2-deoxy[1-^3^H]Glc-6-phosphate

2-deoxy[1-^3^H]Glc-6-phosphate ([1-^3^H]DOG6P) was prepared from [1-^3^H]DOG, and used as internal standard for monitoring the recovery of [U-^14^C]DOG6P from extraction procedures. Twenty µCi of [1-^3^H]DOG (4 Ci mmol^−1^) were added to 100 µL buffer containing 50 mM Tris-HCl, pH 8.0, 50 mM KCl, 20 mM MgCl_2_, 6.5 mM DOG, 20 mM ATP and 0.18 U of commercial yeast HK. The mixture was incubated for 30 min at 37°C, and brought to a 3-mL final volume containing 1 µmol DOG and 1 µmol DOG6P (carriers). The solution was fractionated on Dowex AG 50W-X8 (H^+^) and Dowex AG 1-X8 (formate) as described above. [1-^3^H]DOG6P eluted in the anionic fraction, which was dried under vacuum for 4 h and resuspended in 5% (v/v) ethanol. The overall yield of ([1-^3^H]DOG6P) production from [1-^3^H]DOG was 50%.

### Isotopic Labeling of Roots with [U-^14^C]DOG, [U-^14^C]Glc and [U-^14^C]Suc

Roots grown in liquid MS medium were labeled with [U-^14^C]DOG. Accumulation rates of [U-^14^C]DOG6P were linear for at least 10 h of labeling, indicating that the tissue was at metabolic and isotopic steady state within the time frame used for incubation with the tracer [53]. In order to limit the effects of further metabolism of [U-^14^C]DOG6P noted elsewhere [54], the conversion of [U-^14^C]DOG to [U-^14^C]DOG6P was investigated for up to 8 h after addition of the tracer. Roots (0.25 g FW) were incubated in 18.5 kBq of [U-^14^C]DOG (230–330 mCi mmol^−1^) in a 25 mL Erlenmeyer. Following incubation, roots were extracted in 80% (v/v) ethanol in the presence of 1 µmol of both DOG and DOG6P added as carriers and 1.66 kBq [1-^3^H]DOG6) used as internal recovery standard. Roots were fractionated as described above. The radioactive compound present in the anionic fraction was identified as [U-^14^C]DOG6P based on copurification with the [1-^3^H]DOG6P standard and its copurification with the [U-^14^C]DOG standard following incubation with alkaline phosphatase. To examine the metabolism of [U-^14^C]Glc and [U-^14^C]Suc, roots grown in liquid MS medium were incubated with 37 kBq of tracer in a screw cap Warburg flask which contained 2 mL KOH 1 M in the center well to trap ^14^CO_2_. Following incubation, metabolization of the tracer was examined by counting the radioactivity present in the KOH trap and in the roots which were subjected to ethanol extraction and fractionation as described above. The recovery of radioactivity in labeling experiments was >93%. Labeling of roots with [U-^14^C]Glc or [U-^14^C]Suc led to a linear production of ^14^CO_2_ over the labeling time span. In some experiments, roots were fed with [U-^14^C]Glc as described above except that various incubation times were used. Following incubation, roots were fractionated into neutral, cationic and anionic fractions, and aliquots of the neutral fraction were separated on a Supelcogel Pb column, as described above. The peaks corresponding to Suc, Glc and Fru were separated and collected using a Waters fraction collector and counted for radioactivity.

### Statistical Analyses

Statistical analysis of the data was done using Student’s *t*-test component of SigmaPlot 8.0 (SPSS Inc, Chicago, Il). A value of *P*<0.05 was considered significant. The Pearson correlation coefficient was calculated using Microsoft Excel (Microsoft Corporation, Redmond, WA, USA) and values<−0.5 and >+0.5 were considered significant.

## Results and Discussion

### Generation and Characterization of Root Clones with Differing HK Activity Levels

**Figure 1:**
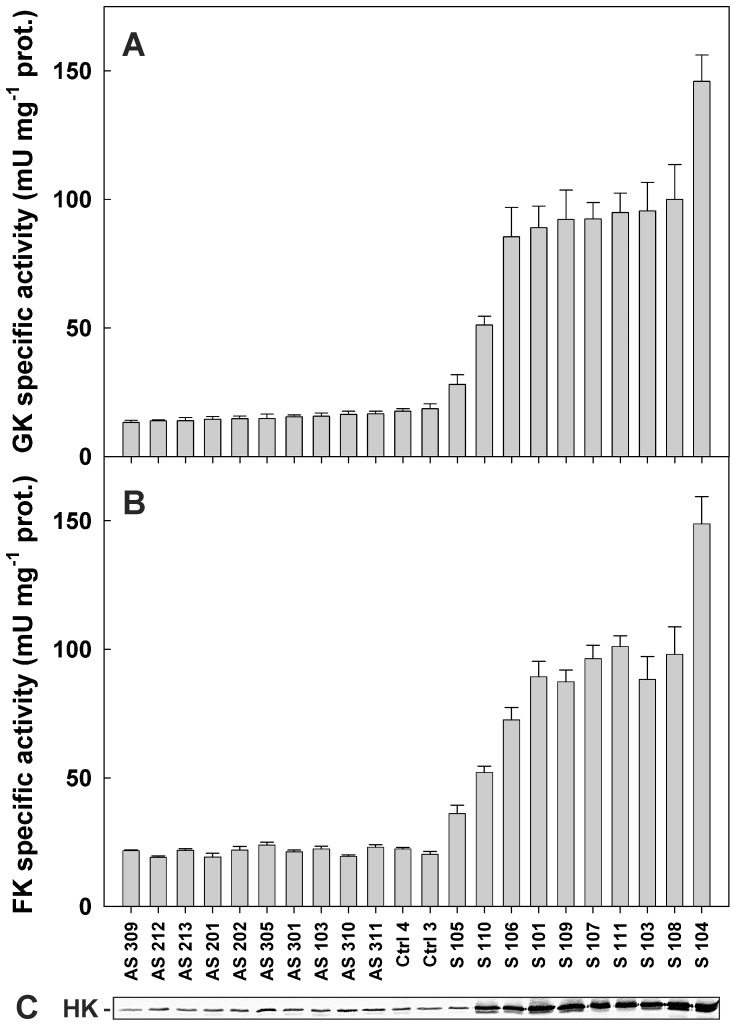
Total extractible HK activity and protein levels in transgenic potato root clones. Specific activities were measured with Glc (GK activity, a) and Fru (FK activity, b) in antisense (AS), control (Ctrl) and sense (S) clones. Values are means ± SE from three to ten separate extractions. Immunoblot analysis (c) of HK protein levels in potato root clones. Immunodetection was carried out with affinity-purified IgGs raised against recombinant ScHK2. Each lane was loaded with 6.5 µg of protein. The names of the clones at the bottom of the bar graph also identify the lanes in panel (c).

We generated a population of root clones with varying HK activity levels to study the control of HK on root growth and on glycolytic flux. One sense and three antisense constructs of the *ScHK2* cDNA [20], inserted in the pGA643 vector under the control of the CAMV 35S promoter, were used for *A. rhizogenes* mediated transformation of potato stems. Depending on the HK construct introduced, the root clones constitutively over- or underexpressed HK. The introduction of *ScHK2* in antisense orientation was expected to efficiently down-regulate HK expression in potato roots since it shared 82–98% overall identity with known potato HK sequences [20]. More than one hundred independent clones were generated using different constructions. This population was screened using HK activity assays and twenty two clones representing the range of activity present in the initial population were selected for further studies. HK activity present in these clones was measured using Glc and Fru as substrates and is represented in Fig. 1a–b. Two control (Ctrl) clones transformed with an empty pGA643 vector served as references for the sense and antisense clones. Ten antisense clones displayed between 72% and 90% of HK activity levels observed for the Ctrl clones. Transgenic roots displaying lower activity levels could not be found in the screening process despite the use of 3 different constructs in the antisense strategy. The simplest explanation for this is that the HK sequences used in our antisense constructs did not have enough homology with *HK* genes expressed in roots to down regulate them efficiently. Ten sense clones had HK activity levels 1.5 to 8 times higher than those of control clones (Fig. 1a). GK and FK components of these HK activities were well correlated (Fig. 1a,b). The root clones were subjected to immunoblot analysis with an anti-HK2 affinity-purified IgGs [20], as shown in Fig. 1c. The differences in HK protein levels detected among the clones followed the general trend of HK activity values (Fig. 1c).

### HK Isoform Profiles of Three Representative Potato Root Clones

Multiple HK and FK isoforms have been described in plants [2], [4], [55]. Hexose-phosphorylating activities present in transgenic potato roots were examined by analytical anion-exchange chromatography (Fig. S1). Protein extracts from representative antisense (AS301), control (Ctrl3) and sense (S111) clones were separated by anion-exchange chromatography to study the impact of sense and antisense strategies on the potato root HK and FK isoform profiles. Two peaks, identified 1 and 2, eluted respectively at fractions 20 and 23 in the GK (Fig. S1B) and FK (Fig. S1E) profiles of clone Ctrl3, indicating the existence of at least 2 HK isoforms in the roots of *S. tuberosum*. A third peak (peak 3) with only FK activity eluted between fractions 30 and 45 (Fig. S1E). Integration of the activity peaks in Ctrl3 indicated that peaks 1 and 2 respectively represented 65 and 35% of total GK activity. With respect to clone AS301, peaks 1, 2 and 3 were observed at the same fractions as Ctrl3 (Fig. S1A, D). Peaks 1 and 2 were both decreased, indicating that the antisense strategy affected both HK isoforms found in Ctrl clones. In clone S111 a single, large peak of HK activity eluted at fraction 23 and hence, was also named peak 2 (Fig. S1C, F). These data are consistent with the fact that ScHK2 was strongly expressed in the S111 clone, and gave rise to a large activity peak engulfing the two preexisting peaks 1 and 2 (Fig. S1C, F).

### Activities of Primary Metabolism Enzymes

The activity of 21 enzymes of primary metabolism was surveyed in two antisense (AS213, AS301), one control (Ctrl3) and two sense (S101, S111) clones (Table 1). Activity values obtained for Ctrl3 were generally comparable to other hairy root cultures [46] and those reported for roots, germinating seeds or suspension cells [56]–[58] [42], [59]–[63]. Only GK and FK activities varied significantly among the 5 clones that were surveyed. It was therefore concluded that the engineered modifications in HK activity did not have a detectable pleiotropic effect on the activity of other enzymes in primary metabolism. Among all the glycolytic enzymes assayed in Ctrl clones, GK was one of the lowest activities. Our data demonstrate that transgenic roots contain INV and Susy activities, which could feed HK with its substrates Glc and Fru. The capacity for hexose-P dephosphorylation was assessed using G6P as a substrate (G6Pase) (Table 1). Under these conditions, hexose-P phosphatase activity was comparable to that of GK activity in Ctrl clone. When G6P was replaced by fructose-6P (F6P) or glucose-1P (G1P), activities were 2.5 and 7 times lower, respectively. The use of a mixture of G6P, F6P and G1P did not increase the reaction rate. It remains to be determined if a single or different enzymes hydrolyze all the hexose-Ps tested as substrates and whether or not this activity is different from previously described enzymes hydrolyzing phosphate esters [64]. Our data nonetheless indicate the existence of activity(ies) capable of dephosphorylating hexose-Ps in crude extracts of roots. In addition to G6Pase, Susy and UGPase acting in tandem could also be involved in cycling hexose-Ps. These enzymes both catalyze reversible reactions and could thus be able to carry out an important flux to Suc [65], [66].The activities of these two enzymes was high compared to GK. Depending on the particular clone considered, the activity of Susy was 2.3 to 17 times higher than GK activity, and that of UGPase was 9 to 65 times superior to HK.

**Table 1:** Specific activities of primary metabolism enzymes assayed in two antisense (AS213, AS301), one control (Ctrl3) and two sense (S101, S111) clones.

	AS213	AS301	Ctrl3	S101	S111
Cell wall INV	170±6	79±10	105±5	111±4	119±4
Acid INV	10±1	9±1	10±2	14±1	18±1
Neutral INV	4±1	4±1	3±1	4±1	4±1
SuSy	238±20	228±30	212±20	210±20	263±30
UGPase	918±170	896±180	698±40	826±75	1033±70
AGPase	20±6	17±6	15±1	16±2	20±4
GK	14±1	15±1	19±2	89±8	95±8
FK	22±1	21±1	24±2	89±6	101±4
PGI	652±30	621±80	596±10	641±30	728±30
PGM	256±10	258±20	245±10	242±10	273±20
G6Pase	19±2	23±2	22±1	24±1	23±1
PFK	65±4	64±4	65±3	64±2	85±4
FBPase	15±1	15±1	15±1	15±1	16±1
PFP	262±10	252±10	262±10	253±10	270±10
ALD	47±1	49±10	46±1	47±1	46±1
TPI	1490±90	1810±40	1660±140	1800±110	1480±60
NAD:GAPDH	1450±140	1400±160	1480±110	1540±60	1550±40
NADP:GAPDH	3±1	2±1	3±1	2±0	3±1
PK	412±50	338±20	370±30	412±20	428±30
PEPC	112±3	72±2	105±4	114±4	95±4
PEPase	142±5	144±7	144±8	147±4	167±8

Data are mean (mU mg^−1^ protein) ± SE of quadruplicates from four to five independent experiments.

### Alteration of HK Activity Markedly Affects Root Growth

**Figure 2:**
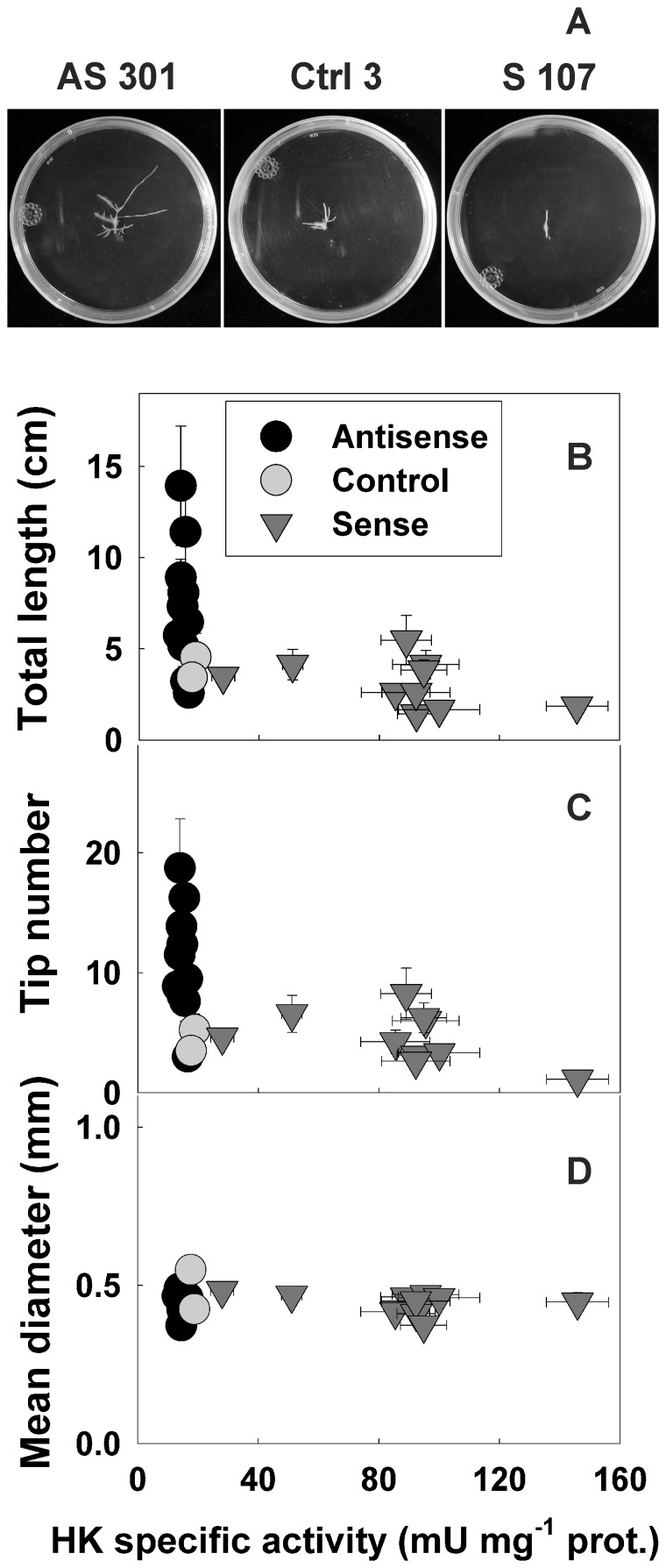
Effects of altered HK activity levels on potato root growth. a Representative pictures of antisense (AS301), control (Ctrl3) and sense (S107) clones grown for 14 d from a single 0.7 cm-long root tip. Total root length (b), tip number (c) and mean diameter (d) were assessed for antisense (black circles), control (grey circles) and sense (grey triangles) clones after 14 d of growth on solid MS medium. Y values are means ± SE of quadruplicates from two independent experiments, X values are from Fig. 1.

We observed pronounced differences in growth among the transgenic root clones. This was illustrated by comparing the growth from a single root tip over 14 d for clones AS301, Ctrl3 and S107 (Fig. 2A). This prompted us to quantify several growth parameters (total root length, number of root tips and mean root diameter) for the clone population after 14 days of culture from a single root tip (Fig. 2B, C, D). The root total length and tip number tended to decrease significantly with rising HK activities (Fig. 2B, C, Table S1). The observed correlations were stronger when considering the whole population instead of only the antisense and Ctrl clones or the sense and Ctrl clones. Antisense clones exhibited larger changes in growth rates than sense clones despite lesser variations from control values of HK activity (Fig. 2A, B). These data may point to a threshold of HK activity below which root elongation may be enhanced, whereas HK activity increases above that threshold were less efficient in inhibiting root growth. Average root diameter did not change significantly in the clone population regardless of HK activity level (Fig. 2D, Table S1). The total length/tip number ratio was also unaffected by HK activity (data not shown), suggesting that the branching pattern of the clones was not influenced by HK. Importantly, there was no evidence of necrosis or senescence in the slowly-growing sense clones, which continued to grow at a steady but slow pace for 10 days after the experiment and could be successfully subcultured thereafter. Therefore, our study provides support for a role of root HK in growth inhibition without senescence. Root growth inhibition by elevated HK activity has been observed in tomato overexpressing *A. thaliana HXK1* [21]. In that case, grafting experiments linked root growth inhibition to a reduction of photosynthetic activity caused by HK manipulation. Such a possibility can be ruled out here since the root clones were devoid of any aerial part and had access to an ample supply of sucrose in the medium. Other transgenic experiments have revealed differences in the importance of HK in heterotrophic metabolism depending on the the tissue considered. For example, the yield and carbohydrate metabolism of potato tuber was mostly unaffected despite a 7- to 8-fold variation in HK activity levels [67], [68]. In contrast, increased HK activity reduced tomato fruit and seed size, possibly due to slightly reduced respiratory rates and ATP/ADP ratios [22]. Therefore, engineered changes in HK activity levels may have quite diverse impacts in different heterotrophic organs. To better understand the importance of HK in root growth, we further evaluated metabolite contents, respiration and carbon fluxes in root clones displaying varying levels of HK activity.

### Root Metabolite Contents

**Figure 3:**
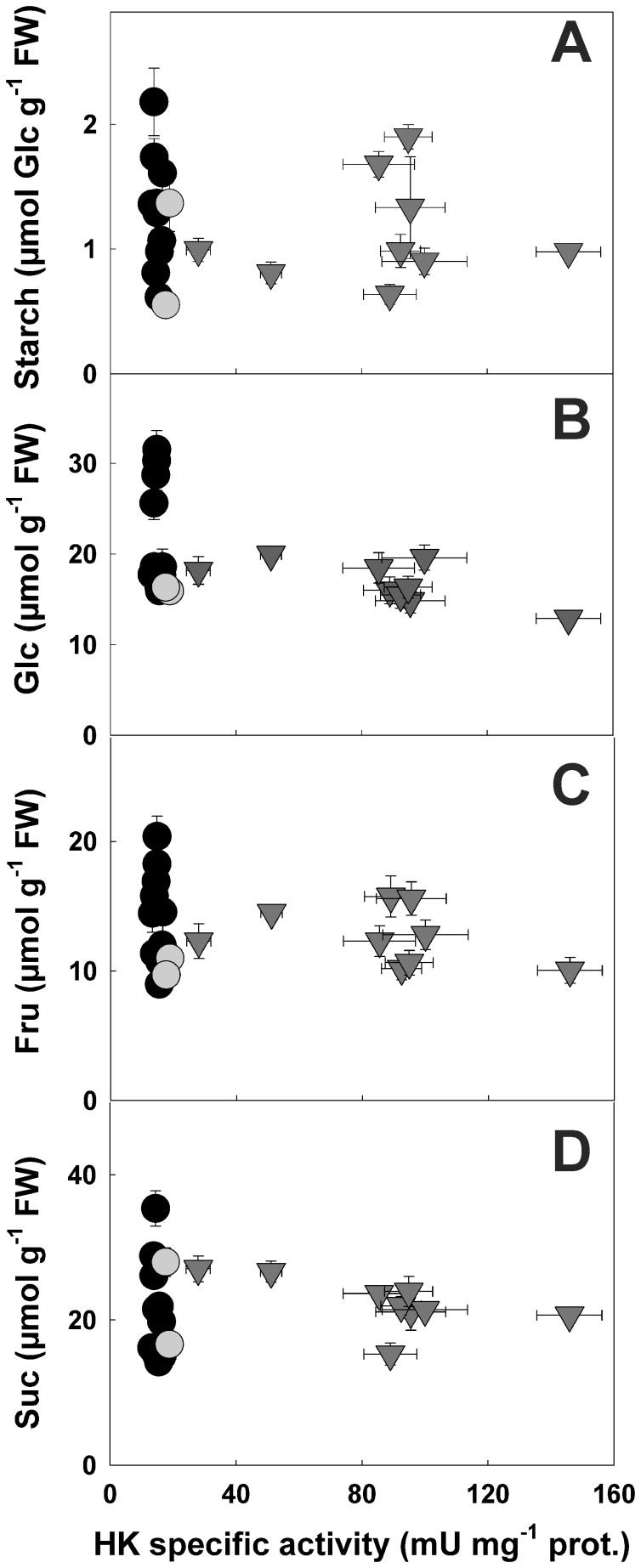
Starch and sugar contents in potato roots with altered HK specific activities. Levels of Starch (a), Glc (b), Fru (c) and Suc (d) were determined in root clones displaying a range of HK activities. Symbols used are: black circles, antisense; grey circles, control and grey triangles, sense clones. Y values are means ± SE from seven to twelve separate experiments, X values are from Fig. 1.

**Figure 4:**
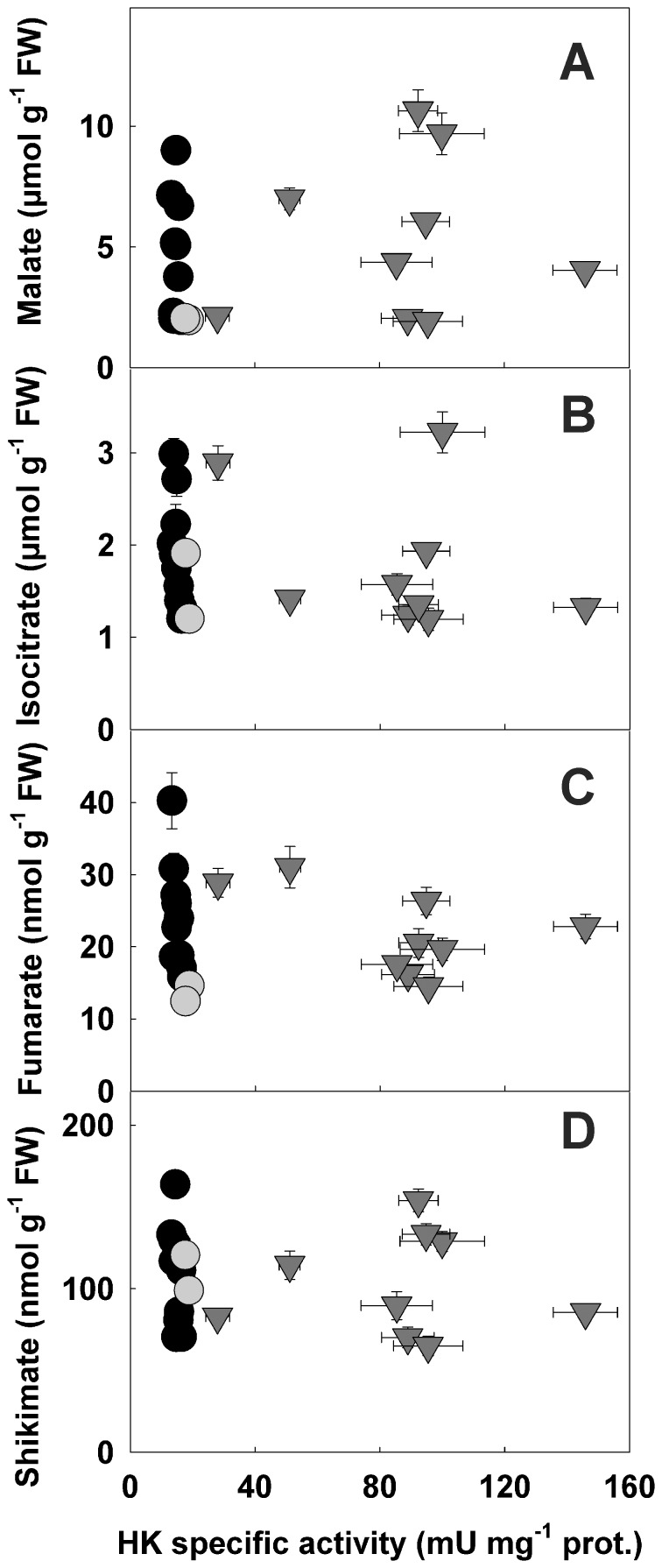
Organic acid contents in potato roots with altered HK specific activities. Levels of malate (a), isocitrate (b), fumarate (c) and shikimate (d) were quantified in root clones displaying a range of HK activities. Symbols used are: black circles, antisense; grey circles, control and grey triangles, sense clones.Y values are means ± SE from seven to twelve separate experiments, X values are from Fig. 1.

**Figure 5:**
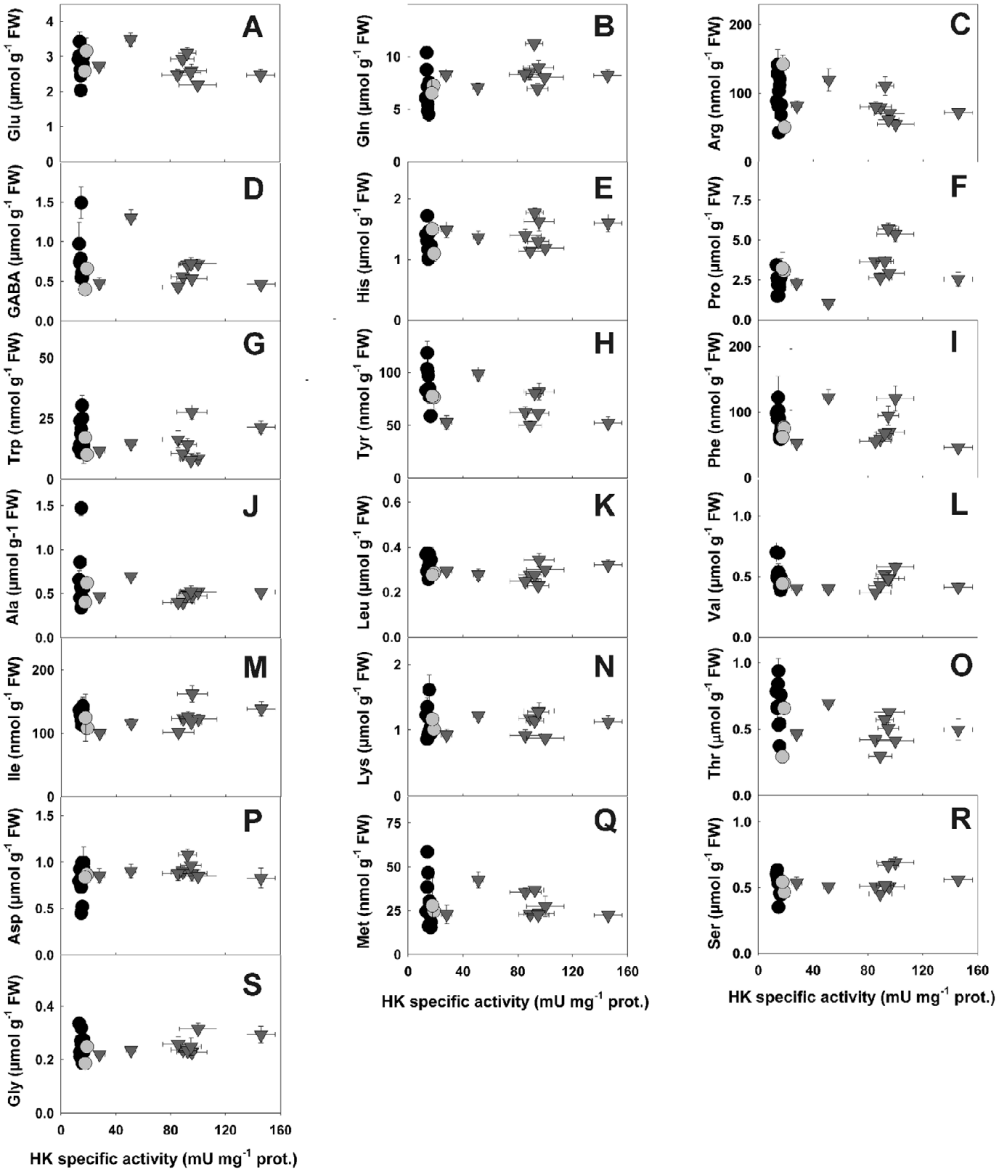
Amino acid contents in potato roots with altered HK specific activities. Levels of Glu (a), Gln (b), Arg (c), γ-aminobutyric acid (GABA) (d), His (e), Pro (f), Trp (g), Tyr (h), Phe (i), Ala (j), Leu (k), Val (l), Ile (m), Lys (n), Thr (o), Asp (p), Met (q), Ser (r) and Gly (s) were determined in root clones displaying a range of HK activities. Symbols used are: black circles, antisense; grey circles, control and grey triangles, sense clones. Y values are means ± SE from seven to twelve separate experiments, X values are from Fig. 1.

The influence of HK on root metabolism was examined by quantifying the pool sizes of major sugars (Fig. 3), organic acids (Fig. 4) and free amino acids (Fig. 5). In the control clones, the measured levels for all these metabolites were in the same range as those normally found in roots [46], [61], [69]. Starch, Fru and Suc levels were not significantly affected by HK levels (Fig. 3A, C, D, Table S1). Tomato fruits overexpressing *A. thaliana* HXK1, showed a decrease in starch levels, particularly during the starch accumulation phase of fruit development [21], [22]. It was concluded that low fruit starch levels in HK overexpressing lines was due to an inhibition of photosynthesis in these plants [21]. In our case, no difference in starch levels was observed in cultured roots with access to a steady supply of Suc from the medium (roots were cultured in liquid MS for 6 d with medium resupply after 4 d). The intracellular pool size of Glc tended to decrease with increasing HK activities, however, the trend was not highly significant (Fig. 3B, Table S1). A Student’s *t*-test indicated that values for Glc contents were significantly different between sense and antisense clones (*P*<0.03). A slight reduction in Glc contents has also been previously observed in potato tubers and tomato fruits overexpressing HK [22], [67].

The major organic acids identified in roots (malate, isocitrate, fumarate and shikimate) were quantified (Fig. 4). No variation in levels of these compounds was significantly correlated to HK activity levels (Table S1). This suggests that the manipulation of HK did not have an impact on the pool size of these downstream metabolites. These results contrast with observations made on tomato fruits overexpressing HK where the levels of malate were shown to increase significantly [22].

Hexose-Ps and adenylates were assayed in two antisense, one control and two sense clones (Table 2). Levels of G6P, F6P and G1P were remarkably similar between the different clones considering their large differences in HK activity. The sum of hexose-Ps levels also remained unaffected by the manipulation of HK levels. Analysis of adenylate pools demonstrated significant differences in ATP and AMP levels in antisense, control and sense clones, leading to a negative correlation between ATP and HK activity levels. These differences in adenylate pools did not affect the sum of adenylate levels but led to higher ATP/ADP ratios and adenylate energy charge (AEC) in antisense clones compared to sense clones. O_2_ uptake was also measured in the same clones. The differences in O_2_ uptake values observed between sense and antisense clones were not significant, indicating that respiratory activity in roots was not affected by the manipulation of HK activity. Tomato fruits overexpressing HK were shown to have significantly lower O_2_ consumption, although the observed differences were not very large [22].

**Table 2:** Measurements of metabolites, adenylates, and O_2_ uptake in antisense (AS213, AS301), control (Ctrl3) and sense (S101, S111) clones.

		AS213		AS301		Ctrl3		S101		S111
Metabolites (nmol g^−1^ FW)										
G6P	470±11		387±15		447±17		486±19		532±18	
F6P	223±13		199±15		238±14		236±11		266±11	
G1P	232±28		196±13		203±12		193±9		264±14	
∑ hexose-P	925±52		782±43		888±43		915±39		1062±43	
ATP	145±7^a^		157±10^a^		125±11^b^		116±11^c^		113±7^c^	
ADP	36±2		37±2		38±3		47±3		36±3	
AMP	8.1±1.5^a^		9.5±1.1^a^		4.7±0.7^b^		12.2±1.6^c^		11.8±1.6^c^	
∑ adenylates	189±10		200±13		168±15		175±16		161±12	
Adenylates ratios										
ATP/ADP	4.0±0.1^a^		4.3±0.3^a^		3.5±0.2^ab^		2.7±0.3^b^		3.5±0.2^b^	
AEC	0.85±0.01^a^		0.86±0.01^a^		0.85±0.01^a^		0.80±0.01^b^		0.80±0.02^b^	
O_2_ uptake (µmol min^−1^ g^−1^ FW)										
	0.98±0.05		1.09±0.07		0.93±0.06		0.84±0.06		0.93±0.05	

Data are mean ± SE for 9 to 20 independent measurements. Values marked with a particular superscript letter denote that they are significantly different from values marked with a different superscript letter on the same line (Student’s *t*-test, *P*<0.05). Values that are not marked with a superscript letter are not significantly different between antisense, control and sense clones.

Glycolytic and respiratory metabolisms provide C skeletons for biosyntheses, in particular for amino acids [1]. We therefore further investigated the possible effects of manipulating HK activity by quantifying the pools of free amino acids in the root clones (Fig. 5, Table S1). Total amounts of N in these amino acids were relatively stable across the entire transgenic population (27.3±0.8 µmol N g^−1^ FW, not shown), indicating that HK manipulation did not have an impact on global N assimilation. There were very little variations in the pools of amino acids as a function of HK activity variation. Only Gln and Pro levels were slightly positively correlated with those of HK activity (Fig. 5B, F, Table S1). Pro is a known marker of stress and its amount may increase in a variety of conditions [70]. The modest increase in Gln levels in HK-overexpressing roots may be related to the low ATP levels, which, in turn, could possibly limit of the ATP-dependent reactions involved in N distribution from Gln (e.g. the Asn synthase (EC 6.3.5.4) reaction). The lack of changes in amino acid metabolism in transgenic roots contrasts with observations made in the fruits of tomato plants overexpressing HK [22]. In this material, the pools of several amino acids, including Gly, Ser, Ala, GABA, were shown to increase, thus doubling the pool size of total free amino acids at several stages of fruit development [22]. The reasons for these differences may lie in the fact that tomato is a climacteric fruit and that, in this particular context, respiration was slightly decreased in fruits overexpressing HK [22]. As seen above, respiration of transgenic roots was unaltered (Table 2).

Representative root clones were analyzed to determine their Pi contents and were subjected to *in vivo* ^31^P NMR analysis to evaluate the compartmentation of the main intracellular Pi pools as well as the pH values of the cytoplasmic and vacuolar compartments (Table 3). A significantly lower Pi content was observed in sense compared to antisense clones. However, this condition was not sufficient to promote Pi starvation and its deleterious effects [1] in HK overexpressing roots. Indeed, these clones showed (i) no increased activity of Pi-stress inducible enzymes [71] such as PEPase and PEPC (Table 1), (ii) no decrease of total adenylates (Table 2) and (iii) no effect on their glycolytic flux (see below). No trend was observed in the allocation of Pi between the vacuolar and the cytoplasmic compartments (Table 4). These data indicate that the observed reduction in Pi content did not trigger a mobilization of stored Pi from the vacuole. Finally, intracellular pH values did not change between clones expressing different levels of HK activity (Table 3). Thus, despite lower ATP levels in sense clones, there was no evidence of an impediment in the function of ATP-dependent H^+^ pumps, which are important mediators of cytoplasmic pH regulation [72].

**Table 3:** Pi pools and intracellular pHs.

	AS213	AS301	Ctrl3	S101	S111
Pi (µmol g^−1^ FW)	12.4±0.3^a^	11.1±0.9^a^	8.9±0.4^b^	9.6±0.7^b^	7.6±0.4^c^
Intracellular Pi pool (%)					
Vacuolar	76.8±1.2	77.4±1.3	76.1±1.3	81.8±1.2	74.4±0.8
Cytoplasmic	23.2±1.2	22.6±1.3	23.9±1.3	18.2±1.2	25.6±0.8
Intracellular pHs					
Vacuolar	5.74±0.08	5.51±0.06	5.41±0.20	5.48±0.43	5.71±0.07
Cytoplasmic	6.77±0.01	6.78±0.01	6.82±0.05	6.89±0.14	6.79±0.01

Pi quantification was done using a colorimetric assay. Intracellular Pi pools and pHs were measured by *in vivo* ^31^P NMR. Root cultures (0.5–0.8 g FW) were inserted into a 10 mm NMR tube perfused with oxygenated MS medium. Spectra were recorded over periods of 1 h on a Varian Unity Inova 400 MHz NMR spectrometer.

**Table 4:** Distribution of radioactivity in different fractions after feeding two antisense (AS213, AS301), one control (Ctrl3) and two sense (S101, S111) root clones with [U-^14^C]Glc.

		AS213		AS301		Ctrl3		S101		S111
Radioactivity recovered in each fraction (%) ± SE										
CO_2_	52.0±2.6		47.3±1.4		43.7±1.3		43.8±2.2		44.8±1.2	
Neutral	12.2±1.1		10.0±0.4		10.6±0.1		13.4±0.6		11.7±0.8	
Anionic	15.3±1.0		18.2±0.9		17.4±0.4		15.5±0.7		13.2±0.3	
Cationic	8.4±0.3		9.4±0.3		9.7±0.3		10.9±0.4		10.6±0.3	
Insoluble	16.9±0.8		17.6±0.9		18.5±1.0		19.7±1.5		19.6±0.8	

Following incubation with the radioactive tracer, radioactive CO_2_ trapped in KOH was counted. Roots were extracted in 80% (v/v) ethanol and the extract fractionated into neutral, acidic and basic fractions. Aliquots of each fraction were counted. The radioactivity in the insoluble fraction represents the ethanol insoluble residue. Data are mean ± SE of 5 to 7 labeling experiments.

Overall, our results demonstrate that the manipulation of HK in roots did not lead to major modifications in the pools of metabolites of glycolytic and respiratory metabolism. The only significant impact was found on the levels of ATP and Pi. In order to further evaluate the function of HK *in vivo*, we carried out a series of metabolic flux measurements with various substrates.

### High Control of HK Over Glc Phosphorylation

**Figure 6:**
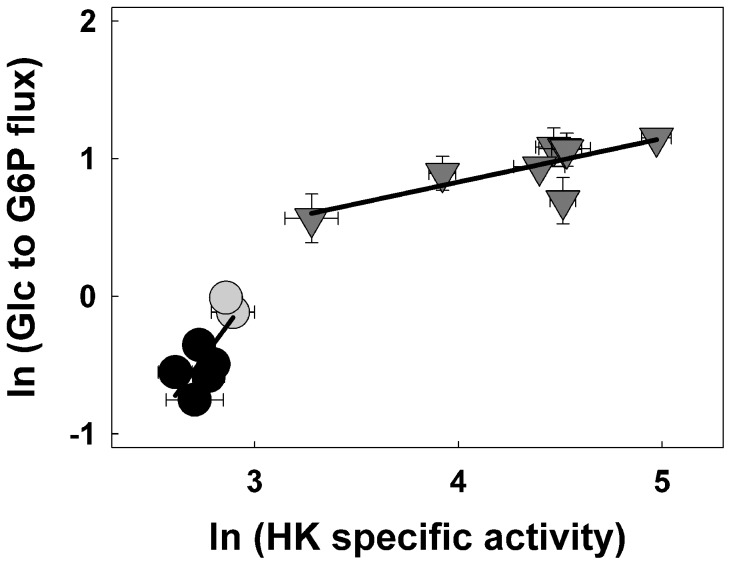
Determination of the flux control coefficient of HK over the phosphorylation of Glc in potato roots. Glycolytic flux from Glc to G6P was quantified in transgenic root clones displaying a range of HK activities. Fluxes were calculated using the Glc analog [U-^14^C]DOG as a tracer. The specific radioactivity of the hexose pool in each labeling experiment was calculated by measuring the radioactivity of the neutral fraction and the pool size of Glc. This specific radioactivity was used to convert the appearance of radioactivity in [U-^14^C]DOG6P to molar fluxes. The flux control coefficient (slope of the regression lines) was calculated for root clones with low HK activity (antisense and contol clones) and high HK activity (sense clones). Symbols used are: black circles, antisense; grey circles, control and grey triangles, sense clones. Y values are means ± SE of triplicates from three to five separate experiments, X values are natural logarithm values of HK activities from Fig. 1.

Glc phosphorylation was measured *in vivo* using the Glc analog [U-^14^C]DOG as a tracer. DOG is taken up by plant cells and phosphorylated by HK into DOG6P. Experimental evidence shows that further metabolization of [^14^C]DOG6P is extremely slow [54]. Various treatments of plant cells with DOG (e.g. 10 h at 0.1 to 1 mM [54], 1 h at 100 mM [73]) have been shown to affect metabolism, possibly due to further metabolization of DOG6P [54]. However, short term incubations with low amounts of [U-^14^C]DOG have been successfully used to examine *in vivo* HK activity in roots [59] as well as in animal cells [74], [75]. Using [U-^14^C]DOG (at 0.4 µM) we were able to show that [U-^14^C]DOG6P levels rose linearly between 0 and 10 h of labeling, and declined thereafter (data not shown). We therefore used this concentration and a labeling time of 2 h to evaluate the Glc-to-G6P flux by quantifying the rates of [U-^14^C]DOG6P accumulation and the specific radioactivity of Glc in samples incubated with [U-^14^C]DOG. The natural logarithm of the Glc-to-G6P flux was plotted against the natural logarithm of HK activity [76] to assess the flux control coefficient (FCC) of HK over Glc phosphorylation (Fig. 6). Examination of the data indicates that the FCC was the highest at or below the normally occurring range of HK activities (antisense and Ctrl values) and was lower at high HK activity values. Such variation is relatively normal [76]. In order to evaluate the range of variation in FCC values, two linear regression analyses were performed (Fig. 6). The remarkably high value of 1.71±0.34 was obtained for clones displaying the lowest HK activity values (antisense and Ctrl roots). This indicates a very high control of HK over Glc phosphorylation at physiological HK activity values. Such a high FCC value could also be interpreted as evidence that around its normal operating point, HK exists an activated state [76]. The FCC value calculated from the sense clones was 0.32±0.03, which is still a high value compared to most enzymes [76]. The average FCC of HK over Glc phosphorylation across the whole population of clones was estimated at 0.76±0.08. To our knowledge, these data provide the first report on the level of control of HK over the phosphorylation of Glc in plants. A high (0.7 to 1) FCC of HK over glycolysis or its upper segment has been reported in various mammalian tissues and cell types [10]–[15], [77]. Our results now provide evidence for similarly high FCCs of HK in both plants and mammals, despite major differences in the structure and regulation of their glycolytic pathways [78]. In yeast, a lower (0.2 to 0.5) FCC has been assigned to HK, which may be due to its inhibition by T6P [18], [19]. It has been proposed that glycolytic regulation by T6P has evolved specifically to cope with sudden and large variations in Glc availability, in contrast to mammals where Glc levels are relatively constant [17]. In plants, only some HK isoforms have been found sensitive to G6P inhibition and none to T6P [2]. Since HK is a major entry point for the glycolytic pathway, we investigated the effects of varying HK activity on the metabolization of Glc and Suc into glycolytic and respiratory pathways.

### Manipulation of HK in Roots does not Alter the Catabolism of Glc or Suc to CO_2_

The metabolization of [U-^14^C]Glc used as a tracer was monitored in representative antisense, control and sense root clones (Table 4). Sense clones tended to have a slightly lower output of ^14^CO_2_ and antisense clones a somewhat lower proportion of C directed to insoluble material (representing cell wall and starch). However, these differences were minor and statistical analyses of the radioactivity metabolized between the different fractions did not show any significant difference in Glc catabolism between clones expressing different levels of HK.

Roots were also labelled with [U-^14^C]Suc, since this sugar was the source of C used in the growth medium. The metabolization of supplied [U-^14^C]Suc was monitored and, consistently with the results of the [U-^14^C]Glc labeling experiment, no difference was observed between the control, sense and antisense clones (Table 5). From these data, a value of 0.02 could be calculated for the FCC of HK over the flux from Suc to CO_2_. This indicates that, even though HK exerts a high control over Glc phosphorylation, HK activity *per se* does not control the catabolism of Suc to CO_2_ in roots. This result also further validates the observations that HK levels did not influence O_2_ uptake (Table 2). As a consequence, it would appear that the strong influence that HK has on *in vivo* Glc phosphorylation does not lead to a corresponding effect on downstream metabolism.

**Table 5:** Distribution of radioactivity in different fractions after feeding an antisense (AS213), a control (Ctrl3) and a sense (S101) root clones with [U-^14^C]Suc.

	AS213	Ctrl3	S101
Radioactivity recovered in each fraction (%)			
CO_2_	49.0±1.1	58.0±1.5	53.3±1.4
Neutral	27.8±0.9	22.1±1.1	23.7±0.2
Acidic	2.6±0.1	1.7±0.1	2.3±0.3
Basic	8.0±0.3	6.8±0.7	7.8±0.3
Insoluble	12.6±1.2	11.3±0.9	12.8±1.2
Flux Suc to CO_2_ (µmol Suc h^−1^ g^−1^ FW)			
	3.6±0.2^a^	4.6±0.2^b^	4.2±0.5^ b^

Following incubation with the radioactive tracer, radioactive CO_2_ trapped in KOH was counted. Roots were extracted in 80% (v/v) ethanol and the extract fractionated into neutral, acidic and basic fractions. Aliquots of each fraction were counted. The radioactivity in the insoluble fraction represents the ethanol insoluble residue. Data are mean ± SE of 3 to 4 labeling experiments.

### Evidence for Futile Cycling between Hexose-Ps and the Free Sugar Pools in Transgenic Roots

**Figure 7:**
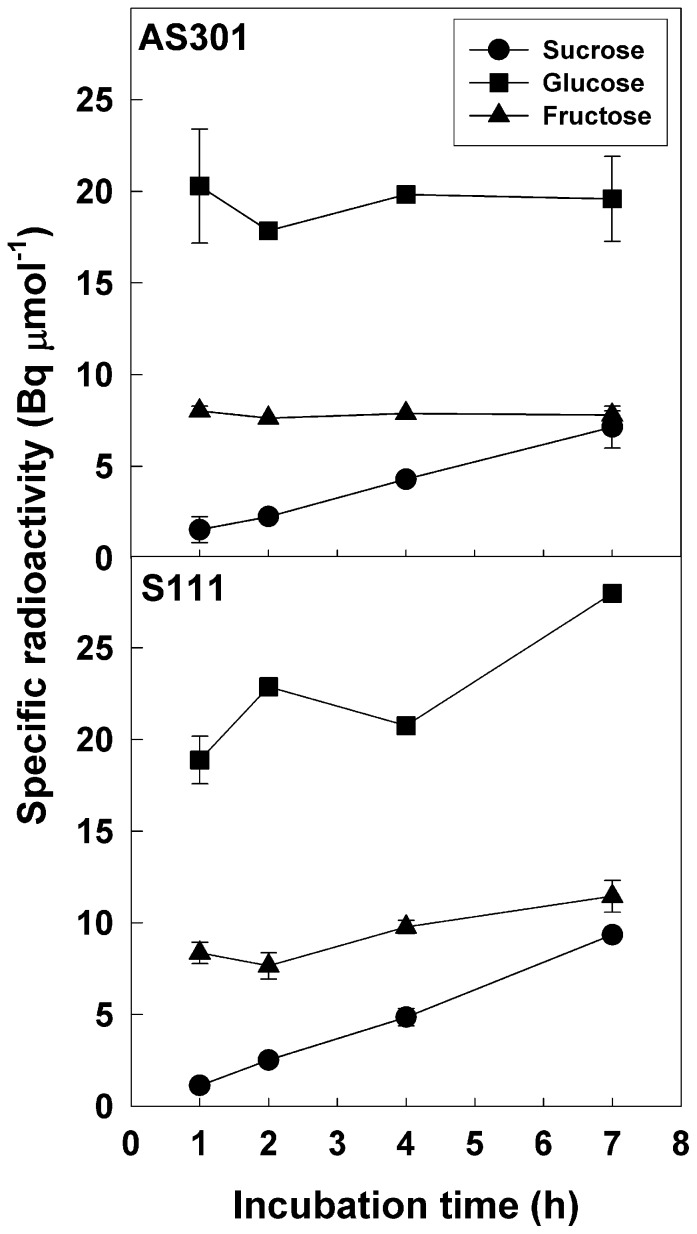
Evidence for futile cycling of glucose in transgenic roots. An antisense (AS213) and a sense (S111) root clones were incubated for various time durations with [U-^14^C]Glc. Following incubation with the radioactive tracer, roots were extracted in 80% (v/v) ethanol and the neutral fraction was purified by negative ion exchange chromatography. An aliquot of each neutral fraction was subjected to HPLC on a Supelcogel Pb column to separate the peaks of Glc, Fru and Suc which were collected. The specific radioactivity of each sugar present in the two clones after various labeling times was calculated by integrating the HPLC profile corresponding to sugar peak and quantifying the radioactivity present in the peak collected after HPLC. Data representative of three different labeling experiments are shown.

Futile cycles involved in the interconversion of the pools of (i) hexose-Ps and hexoses and (ii) hexose-Ps and Suc have been detected in non-photosynthetic plant tissues [25]. The occurrence of substrate cycling involving hexose-Ps formed by HK to the pools of free hexoses and Suc would be consistent with the above results, because such cycling would dampen the net flux from hexoses to hexose-Ps, at the expense of ATP. Our data show that fed [U-^14^C]Glc can be efficiently phosphorylated to [U-^14^C]G6P (Fig. 6) and further metabolized to ^14^CO_2_ (Table 4). We have also demonstrated the presence of a catalytic potential for the dephosphorylation of G6P (Table 1), enabling the return of carbon from G6P to the Glc pool. In order to further investigate the occurrence of substrate cycling *in vivo*, antisense and sense roots were labeled with [U-^14^C]Glc for various time durations. Following incubation, we isolated Suc, Glc and Fru by collecting the neutral fraction after ion exchange chromatography and subjecting it to HPLC separation. The peaks corresponding to these 3 carbohydrates were quantified, collected and their specific radioactivity was determined (Fig. 7). In these analyses, the peaks of Suc, Glc and Fru were the only ones where radioactivity was found and signal integration of the refractive index detector indicated that they represented more than 95% of the total sugars present in the neutral fraction. No significant difference was observed between labeling results of antisense and sense clones, indicating that the manipulation of HK did not have an impact on the specific radioactivity of the sugars. However, distinct patterns of labeling were apparent between Suc, Glc and Fru. Compared to the other sugars, the specific radioactivity of Glc was always the highest. This can be expected, since roots were fed with this tracer. Fru was also labeled and its specific radioactivity was stable or slightly increased between 1 and 7 h of labeling. The possibility of a direct generation of Fru from Glc by a xylose isomerase (EC 5.3.1.5) can be eliminated since (i) higher plant xylose isomerase is unable to use Glc as substrate [79] and (ii) isomerisation between Glc and Fru has not been detected in roots [25]. If we assume that the pool of hexose-Ps is rapidly equilibrated, then, the appearance of labeled Fru may arise from the dephosphorylation of F6P by a hexose-P phosphatase detected in roots (Table 1). It may also occur from the hydrolysis of labeled Suc synthesized from Glc (see below). The specific radioactivity of the Suc pool increased almost linearly over time (Fig. 7). Assuming equilibration of the pool of hexose-Ps, the pattern of labeling of Suc is compatible with the operation of Suc-biosynthesis pathway(s) in transgenic roots. Radioactive Suc can be synthesized from G1P and Fru by UGPase and Susy, which are both highly active in roots (Table 1). It may also occur from G1P and F6P using UGPase combined to the activities of Suc P synthase (EC 2.4.1.14) and Suc P phosphatase (EC 3.1.3.24) which have been detected at low levels in roots [80], [81]. In turn, labeled Suc can be hydrolyzed to Glc and Fru by INV activities detected in roots (Table 1). Another mechanism potentially involved in hexose-P turnover is starch synthesis and degradation [82]. The pathways involved in leaf starch turnover have been recently reviewed [82]. Starch synthesis consumes G1P and its degradation produces mainly the neutral sugars Glc and maltose and small amounts of G1P. Assuming these pathays are active in roots, they would participate in the return of C from the hexose-P pool to the free sugar pool. Interestingly, a model of carbohydrate metabolism in sugar cane predicted a control coefficient of 1 for HK over futile cycling of Suc [66]. However, this model did not take into account the possibility of hexose-P dephosphorylation. The ratios of the Glc/Fru specific activities remained constant at around 2.5 irrespective of the labeling time. This provides an indication that both pools equilibrated rapidly in roots. It remains to be seen if cycling occurs preferentially by the pathway involving hexose-P dephosphorylation [25],through the Suc pool, or by starch turnover. Regardless of the route used for cycling, the net effect of this process would be to decrease the availability of hexose-Ps for downstream glycolytic and respiratory metabolisms. In the sense clones, higher amounts of HK activity did not translate into an increase of the glycolytic flux and hexose-P pools remained unchanged. Cycling could thus be involved in glycolytic regulation by maintaining a stable pool of hexose-Ps.

### Importance of a Tight Control Over G6P Levels

The regulation of the steady state level of G6P may be of prime importance for the maintenance of homeostasis in glycolytic and N metabolisms. Firstly, the inherent risk of Glc-accelerated death due to the autocatalytic principle of the glycolytic pathway may not only apply to yeast, but also to other eukaryotes [17], [83]. A potent inhibition of mammalian HKs I–III by G6P has been proposed to avoid the risk of P_i_ sequestration in hexose-Ps and subsequent Glc-accelerated death in most mammalian cell types [6], [83]. In hepatocytes, the predominant HK isoform is HK IV (‘glucokinase’), which is not product-inhibited by G6P. The ‘guard at the gate’ of hepatic glycolysis may then be provided by Glc/G6P cycling and by HK IV inhibition by a glucokinase-regulatory protein (GKRP) [83]. Combined Suc and hexose/hexose-P cycling may be the plant alternative to T6P-, G6P- and GKRP inhibitions of yeast and mammalian HKs, respectively, for avoidance of Glc-accelerated death by P_i_ sequestration in hexose-Ps [17], [25], [83]. Our results show that although free cytosolic Pi levels were reduced in roots overexpressing HK (Table 3), this was not sufficient to reduce respiration (Table 2) or glycolytic flux (Table 5). Secondly, in addition to the above considerations, Suc and hexose/hexose-P cycles may fulfill another primordial role in plants by adjusting levels of potent regulatory glycolytic intermediates, such as G6P, to hexose supply and downstream metabolic activity. G6P is a key regulator of primary metabolism in plants. In particular, it is known to inhibit SNF1-related protein kinase 1 (SnRK1) [84] and to activate PEPC [85]. These enzymes control important aspects of carbon and nitrogen metabolisms. The metabolite profiles in our transgenic roots suggest that their carbon and nitrogen metabolisms were not deeply affected by the manipulation of HK. Therefore, the hexose/hexose-P and Suc cycles may have efficiently dampened HK activity variations in the root clones. However, the dissipation of ATP by substrate cycling in the sense clones may have led to down-regulation of several processes to reduce ATP expenditure, with possible adverse effects on growth (Fig. 2). It thus appears that in roots, variations in HK activity alone cannot dramatically alter glycolytic flux beyond the hexose-P pool. Our results agree with findings from previous investigations showing that the manipulation of HK activity in potato tubers had limited impact on tuber metabolism [67], [68]. Some differences were apparent between some of our results and those obtained with tomato fruits overexpressing HK [22]. As noted above, this may be due to the particular metabolic context of the climacteric fruit.

In conclusion, we showed that growth was negatively affected in transgenic roots displaying an 11-fold range of HK activity. Compared to antisense clones, root clones with high HK activity had lower levels of ATP, lower ATP/ADP ratio and low AEC values. Examination of the *in vivo* C flux through HK demonstrated a high control of HK over Glc phosphorylation. However, control over glycolysis and respiration was negligible, indicating that utilization of G6P was tightly controlled in roots. Isotopic labeling of roots with [U-^14^C]Glc demonstrated that, while the main fate of Glc was its consumption by glycolysis and respiration, there was formation of radioactive Fru and Suc. These data provide support for the existence of futile cycling involving dephosphorylation of hexose-Ps and/or synthesis/degradation of Suc. Enzyme activities capable of carrying out these reactions were detected in roots. We propose that futile cycling of glucose-6P could be partly responsible for the detected differences in energetic status and growth in roots with high and low HK activity. We also suggest that substrate cycles are necessary to adjust G6P levels to hexose supply and downstream glycolytic activity, and to avoid a stall in glycolytic activity due to sequestration of P_i_ in hexose-Ps. Future elucidation of the mechanisms underlying the regulation of the Suc and hexose/hexose-P cycles will probably help gain further insight into their functions and into the regulation of plant glycolysis.

## Supporting Information

Figure S1 Analytical separation of HK and FK isoforms in desalted extracts of AS301 (panels A,D), Ctrl3 (panels B,E) and sense (S111) (panels C,F) clones, using anion-exchange chromatography. Proteins (2 mg) were loaded on a 6-mL DEAE Fractogel column, and eluted using a KCl gradient (*straight line*). The elution profiles (*filled circles*) of GK (a–c) and FK (d–f) activities are plotted as functions of the fraction number (1 mL per fraction). Activity peaks 1, 2 and 3 are described in the text.
(PDF)

Table S1 **Pearson correlation coefficients between HK activity levels and growth measurements or metabolite pool sizes in the transgenic population.** Unless indicated otherwise, the correlation studies were done on the whole population (AS, Ctrl, and S clones). The notations (AS and Ctrl) and (Ctrl and S) indicates that only clones of these subpopulations were included in the calculation of the corresponding Pearson correlation coefficient.
(PDF)
