# Supplementary figures and images for: The Futile Cycling of Hexose Phosphates Could Account for the Fact That Hexokinase Exerts a High Control on Glucose Phosphorylation but Not on Glycolytic Rate in Transgenic Potato (Solanum tuberosum) Roots
Source: PLoS One. 2013 Jan 28;8(1):e53898. doi: 10.1371/journal.pone.0053898 (PMC3557296; doi:10.1371/journal.pone.0053898)

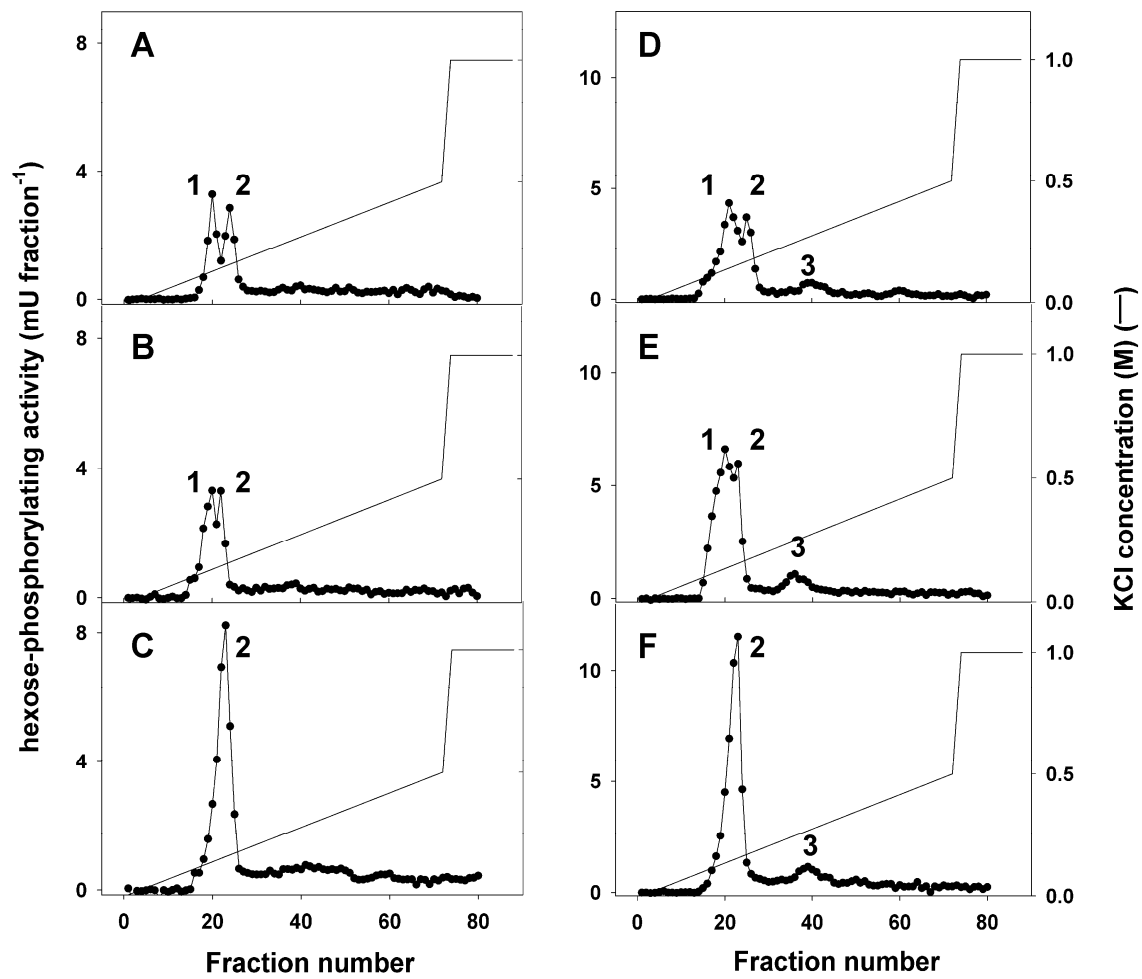

Fig. S1.

Supplement: Figure S1 — Analytical separation of HK and FK isoforms in desalted extracts of AS301 (panels A,D), Ctrl3 (panels B,E) and sense (S111) (panels C,F) clones, using anion-exchange chromatography. Proteins (2 mg) were loaded on a 6-mL DEAE Fractogel column, and eluted using a KCl gradient (straight line). The elution profiles (filled circles) of GK (a–c) and FK (d–f) activities are plotted as functions of the fraction number (1 mL per fraction). Activity peaks 1, 2 and 3 are described in the text. (PDF) [file pone.0053898.s001.pdf]
