# Supplementary material for: The Futile Cycling of Hexose Phosphates Could Account for the Fact That Hexokinase Exerts a High Control on Glucose Phosphorylation but Not on Glycolytic Rate in Transgenic Potato (Solanum tuberosum) Roots
Source: PLoS One. 2013 Jan 28;8(1):e53898. doi: 10.1371/journal.pone.0053898 (PMC3557296; doi:10.1371/journal.pone.0053898)
Supplement: Table S1 — Pearson correlation coefficients between HK activity levels and growth measurements or metabolite pool sizes in the transgenic population. Unless indicated otherwise, the correlation studies were done on the whole population (AS, Ctrl, and S clones). The notations (AS and Ctrl) and (Ctrl and S) indicates that only clones of these subpopulations were included in the calculation of the corresponding Pearson correlation coefficient. (PDF) [file pone.0053898.s002.pdf]

Table S1

Parameter	Pearson correlation coefficient
Growth measurements	
length	- 0.5374
(AS and Ctrl)	- 0.3831
(Ctrl and S)	- 0.3956
tip number	- 0.5514
(AS and Ctrl)	- 0.3678
(Ctrl and S)	- 0.4653
diameter	- 0.1914
(AS and Ctrl)	- 0.1270
(Ctrl and S)	- 0.3408
Metabolites	
starch	- 0.0253
glucose	- 0.4193
fructose	- 0.1769
sucrose	- 0.2945
malate	+ 0.2526
isocitrate	- 0.1279
fumarate	- 0.0987
shikimate	- 0.0412
glutamate	- 0.2568
phenylalanine	- 0.2045
aspartate	+ 0.2791
threonine	- 0.3846
serine	+ 0.2753
glycine	+ 0.1946
alanine	- 0.2025
glutamine	+ 0.4719
histidine	+ 0.3295
proline	+ 0.4971
arginine	- 0.2581
4-aminobutyrate	- 0.1757
tyrosine	- 0.0828
valine	- 0.1814
leucine	- 0.2227
tryptophan	- 0.0802
isoleucine	+ 0.1719
methionine	- 0.0230
lysine	+ 0.0715
